# Change in body weight and body image in young adults: a longitudinal study

**DOI:** 10.1186/s12889-015-1579-7

**Published:** 2015-03-06

**Authors:** Gicele Costa Mintem, Denise Petrucci Gigante, Bernardo Lessa Horta

**Affiliations:** Postgraduate Program in Epidemiology, Federal University of Pelotas, Brazil Marechal Deodoro, 1160, 96020–220, Pelotas, RS Caixa postal: 354 Brazil

**Keywords:** Body image, Longitudinal studies, Body mass index, Young adults

## Abstract

**Background:**

The goal of this study was to identify the effect of the change in body mass index (BMI) from childhood to adulthood on body image satisfaction at 23 years of age in members of the 1982 Pelotas Birth Cohort in Pelotas, RS, Brazil.

**Methods:**

The study used data from the 1986 and 2004–5 follow-up studies. Body shape satisfaction was evaluated using the Stunkard scale. Body shape dissatisfaction was defined as the difference between the figures chosen for the current and ideal body size. BMI z-score changes were calculated as the difference between z-score values at 4 and 23 years of age, using the population internal z-score as standard. The analysis was stratified by sex, and multinomial logistic regression was used in crude and adjusted analyses.

**Results:**

A total of 1963 men and 1739 women were analyzed. The mean age of the participants in 2004–5 was 22.7 years. Of the participants exhibiting increased BMI z-scores, 17% perceived themselves as thinner than ideal, whereas 48% perceived themselves as fatter than ideal. The prevalence of dissatisfaction was higher in women because they perceived themselves as fatter than ideal on the three categories of z-score change (≥ + 0.5 sd; −0.49 to + 0.49 sd and ≤ −0.5 sd); 81% of women exhibiting an increased BMI z-score reported dissatisfaction. The analysis adjusted for confounding factors revealed that women with increased BMI z-scores were less prone to feel thinner than ideal. Additionally, the increased risk of dissatisfaction due to perceiving oneself as fatter than ideal was similar between men and women (RRR = 3.52 95% CI: 2.17 to 4.56 and RRR = 4.08 95% CI: 3.00 to 5.56, respectively) using −0.49 to +0.49 sd as the reference category.

**Conclusions:**

Individuals exhibiting increased BMI z-scores between 4 and 23 years of age reported higher risks of body dissatisfaction at 23 years of age. This finding is important because body dissatisfaction can cause psychological, social, self-esteem problems, and well-being.

## Background

The concerning worldwide obesity epidemic has prompted research into preventive and effective strategies for promoting and maintaining good health conditions and adequate nutrition [1,2]. The Brazilian nationwide survey entitled The Surveillance of Risk and Protective Factors for Chronic Diseases through Telephone Survey study (VIGITEL) [3] from the Health Ministry reveal that more than half of the Brazilian population is overweight, interviewed 45,000 adults, and 51% of the interviewees mentioned excess weight. The prevalence of obesity was approximately 11% for both sexes in the first edition of the study in 2006, and in 2012, 16% of men and 18% of women classified themselves as obese [3]. The growing trend towards excess weight has raised interest in studies of the body mass index (BMI) trajectories through individuals’ lives [4,5].

In a life cycle perspective, some longitudinal studies suggest that children with excess weight are at greater risk of becoming obese adults [6-8]. According as, obesity in adulthood increases the risk of cardiovascular diseases, some types of cancer, and orthopedic and sleep disorders [9,10]. Many of the negative effects of excess weight in adulthood, such as emotional and psychological problems, negative self-image, decreased self-confidence, depression, social isolation, discrimination, difficulties in interpersonal relationships and eating disorders result from obesity during childhood and the teenage years [6,11-13].

Similarly to excess weight, a high prevalence of body image dissatisfaction, particularly among women, has been reported in several countries [14-18]. Body image dissatisfaction may result in problems of self-esteem, well-being, social group acceptance, job opportunities, productivity, socioeconomic status, and psychosocial performance [19,20]. Body dissatisfaction is central to the health and well-being of children and adolescents who are overweight. In adolescents with high levels of body dissatisfaction, the association between obesity and psychosocial impairment was found [21].

Studies have investigated the relationship between body image and BMI in children and young adults [22-25], but few studies have examined the influence of changes in BMI from childhood to adulthood on body image in young adults, more specifically in Brazil.

Several studies that evaluate BMI trajectories from childhood to adulthood have been published [4,5]. However, only two longitudinal studies of the effects of BMI changes throughout life on body image perception in adulthood could be found in the literature [26,27]. In Holsen et al. [26] study, BMI predicts body image over time for both genders with the exception of early adolescent girls. BMI was important for body image development as the adolescents grow into adults. The second study showed larger decreases in body satisfaction among those whose BMI increased over the 5-year period, except in older adolescent females [27]. In both studies, as expected, the prevalence of body satisfaction was higher in men.

The goal of the present study was to identify how changes in BMI from childhood to adulthood influenced body image satisfaction at 23 years of age in subjects from a cohort study who have been followed-up since birth.

## Methods

The 1982 birth cohort included all infants born alive at hospitals in Pelotas, whose mothers lived within the city’s urban area. Pelotas is a city in southern Brazil, in the State of Rio Grande do Sul, 250 km south of the capital Porto Alegre. The estimated population in 2004 was about 340,000 inhabitants. The main economic activities are agriculture (rice farming and cattle-raising), commerce, and education. Over 90% of the households are connected to the public water supply and half to the sewage disposal network. The population descends mostly from Portuguese and Spanish, Amerindians, Africans, and German immigrants are also represented.

Immediately following childbirth, the mothers answered standardized questionnaires containing demographic, socioeconomic, and health information and were weighed and measured. The infants were weighed. Several follow-ups of the cohort were performed during childhood, adolescence, and adulthood. Further details on the methods used in this study have been previously reported [28,29].

The present study used data from the 1986 and 2004–5 follow-ups. In 1986, 4747 children were evaluated, and in 2004–5, 4297 members of the 1982 cohort were interviewed. A census was conducted in all of the households in Pelotas during both follow-ups. The average age of the members was 43 months in 1986 and 22.7 years in 2004–5. Data relative to socioeconomic status, demographics, environment, and eating were recorded during the interviews.

Other sociodemographic variables (sex, mother’s educational level and family income) were analyzed to identify possible differences in the samples studied in the respective follow-ups. Anthropometric data (weight and height) of the children at four years of age was used to calculate the body mass index (BMI), which was then analyzed using the software WHO-Anthro [30].

During the 2004–5 follow-up, when the evaluation of body satisfaction was performed, 4297 of the 5914 cohort participants were interviewed, and 4100 answered questions concerning body image.

**Figure 1:**
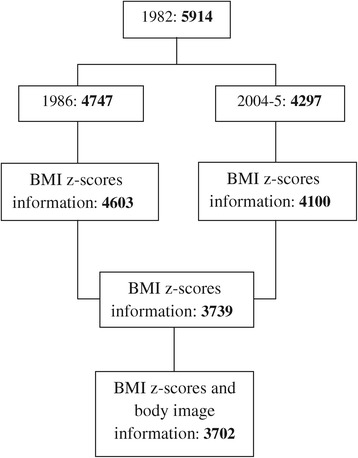
**Flow diagram of sample description.**

The change in body weight between 4 and 23 years of age and its effect on body image dissatisfaction were evaluated in 3702 participants; in addition to answering questions about body image, the participants must have been measured and weighed during the 1986 and 2004–5 follow-ups. A total of 1963 men and 1739 women were included in the study (Figure 1).

For the analysis of changes in BMI, values obtained at 4 and 23 years of age were converted into z-scores using the internal value of the population studied as the standard. The change in BMI z-score was calculated by subtracting the z-score at 4 years of age from the z-score at 23 years of age. A difference of equal or greater than **+** 0.5 standard deviation (sd) and equal or less than −0.5 sd over the time interval studied represents an increase and a decrease in BMI z-scores, respectively, at 23 years of age.

Two questions were used to define outcomes: “Which of these figures do you identify most with your body?” (current body size), and “Which of these figures is closest in appearance to what you would like your body to be?” (ideal body size). To answer these questions, the nine figures composing the Stunkard scale [31] for each sex were presented to the individuals. Participants were asked to choose one figure for each question. Differences between the figures chosen for current and ideal body size were considered to represent dissatisfaction, i.e., the participant perceived him/herself as thinner or fatter than ideal. The use of a validated scale, easy and fast application, seems an appropriate way to identify how individuals perceive themselves in relation to their body.

A statistical analysis was performed to investigate the association between changes in body weight from 4 to 23 years of age and body size satisfaction in adulthood. The analysis was stratified by sex, and multinomial logistic regression was used for the crude and adjusted analyses.

The following socioeconomic variables were included in the analysis adjusted to control for possible confounding factors: mother’s educational level, factorial asset index in 1984 [32], and family income at birth.

In a model adjusted for mediating factors, the following behavioral variables were included: having children at 23 years of age, marital status (without and with a partner), work during the month prior to the interview, psychological well-being (Andrews’s Faces Scale, which scores higher well-being as one to four points and lower well-being as five to seven points), insufficient physical activity (score lower than 150 minutes of physical activity per week according to the International Physical Activity Questionnaire (IPAQ) – long version), smoking (consumption of at least one cigarette per day), and presence of minor psychiatric disorders evaluated according to the SRQ-20 (Self Report Questionnaire-20, with a cut-off point equal to or higher than six for men and equal to or higher than eight for women).

The database included the individuals who were measured and weighed on both follow-ups, in accordance with the exclusion criteria established for the 2004–5 follow-up (physically and/or mentally disabled, Down syndrome, and women who were pregnant or had given birth up to three months prior to the interview), for a total of 3739 individuals. The analyses were conducted using the STATA 12.0 (StataCorp, College Station, TX, USA).

As there was not information about weight status at other ages for all cohort members, we conducted these analysis with men followed from 18 to 23 years.

The 2004–5 follow-up study of the 1982 Pelotas Birth Cohort was approved by the Ethics in Research Committee of the School of Medicine of the Federal University of Pelotas (Universidade Federal de Pelotas - UFPel) (020/2003) and all participants signed an informed consent form. In 1982, only the verbal consent of the mothers was obtained, following the procedure routinely adopted in research at the time.

## Results

At baseline, 11 (0.3%) children were underweight and 1184 (32.0%) had excess weight (overweight and obesity). In 2004–5 follow-up, these values were 230 (6.2%) and 1052 (28.4%), respectively.

No differences between the original cohort and participants located at follow-ups were observed for sex, mother’s educational level, family income in 1982, factorial asset index in 1984, or BMI z-scores at 4 years old (Table 1).

**Table 1:** **Characteristics of the 1982 Pelotas birth cohort in four time points**

**Variables**	**Follow-up studies**			
	**1982 (N = 5914)**	1984 (N = 4934)*	1986 (N = 4742)*	**2004-5 (N = 3702)**
	**n(%)**	**n(%)**	**n(%)**	**n(%)**
**Gender**		*0.83* ^*a*^	*0.87* ^*a*^	*0.11* ^*a*^
Male	3037 (51.4)	2524 (51.2)	2428 (51.2)	1963 (53.0)
Female	2876 (48.6)	2410 (48.8)	2314 (48.8)	1739 (47.0)
**Maternal schooling (years)**		*0.63* ^*a*^	*0.68* ^*a*^	*0.73* ^*a*^
0 – 4	1960 (33.2)	1563 (31.7)	1511 (31.9)	1197 (32.4)
5 – 8	2454 (41.5)	2095 (42.5)	2012 (42.5)	1578 (42.7)
9 – 11	654 (11.1)	559 (11.3)	537 (11.3)	407 (11.0)
≥12	839 (14.2)	712 (14.5)	677 (14.3)	516 (14.0)
**Family income (minimum wages)**		*0.50* ^*a*^	*0.56* ^*a*^	*0.97* ^*a*^
≤1	1288 (21.9)	987 (20.1)	939 (19.9)	716 (19.4)
1.01 – 3.0	2789 (47.4)	2388 (48.6)	2308 (48.9)	1837 (49.8)
3.1 – 6.0	1091 (18.5)	956 (19.5)	915 (19.4)	705 (19.1)
6.1 – 10.0	382 (6.5)	310 (6.3)	299 (6.3)	220 (5.0)
>10.0	335 (5.7)	274 (5.6)	262 (5.6)	209 (5.7)
		Mean (standard deviation)		
**Factorial asset index (1984)**				*0.30* ^*b*^
		3.30 (1.23)		3.32 (1.21)
**Body mass index z-score (4 years old)**				*0.90* ^*b*^
			0.63 (1.01)	0.63 (0.98)

*Participants with height and weight measured ^a^Chi-square test ^b^ *T* test.

Pelotas, Rio Grande do Sul state, Brazil. 1982-2004-5.

The distribution of body dissatisfaction according to changes in body weight between 4 and 23 years of age is presented in Table 2. Almost one third of the all participants decreased or increased BMI z-scores. It is noteworthy that among those who remained in the same category BMI z-scores, about 30% of men perceived themselves as thinner, and 20% as fatter than ideal. Otherwise, in women, 18% perceived themselves as thinner, and about 50% as fatter than ideal. For male participants exhibiting increased BMI z-scores between 4 and 23 years of age, 17% reported that they perceived themselves as thinner than ideal, whereas 48% perceived themselves as fatter than ideal. It is important to note that for the participants exhibiting decreased BMI z-scores between 4 and 23 years of age, 41% perceived themselves as thinner than ideal and 17% perceived themselves as fatter than ideal (Table 2).

**Table 2:** **Body perception according to changes in body weight between 4 and 23 years of age in the 1982 Pelotas birth cohort**

**Variables**	**n**	**Body perception**		
		**Thinner (%)**	**Satisfied (%)**	**Fatter (%)**
MEN				
**Body mass index z-score change**				*<0.001* ^*a*^
≥ +0.5	676	17.2	35.2	47.6
−0.49 to +0.49	755	29.8	51.1	19.1
≤ −0.5	532	40.8	42.5	16.7
WOMEN				
**Body mass index z-score change**				*<0.001* ^*a*^
≥ +0.5	541	4.8	13.9	81.3
−0.49 to +0.49	712	18.1	32.4	49.4
≤ −0.5	486	21.0	31.9	47.1

^a^Chi-squared test.

Pelotas, Rio Grande do Sul state, Brazil, 1982 to 2004–5.

The prevalence of dissatisfaction was observed to be higher for women because they perceived themselves to be fatter than ideal for all three z-score change categories. Among women exhibiting a difference in BMI z-scores ≥ + 0.5 sd, more than 80% reported body dissatisfaction (Table 2). In addition, among those who decreased BMI z-scores 47% perceived themselves as fatter than ideal. All analyses were statistically significant (*p* < 0.001).

The crude results of the multinomial regression revealed strong correlations between changes in z-score and the perception of body shape. Individuals exhibiting increased BMI z-scores between 4 and 23 years of age exhibited a lower probability of feeling thinner than ideal and nearly fourfold greater dissatisfaction due to feeling fatter than ideal. These results were observed for both sexes (Table 3)

**Table 3:** **Crude and adjusted analysis of body perception according to changes in body weight between 4 and 23 years of age in the 1982 Pelotas birth cohort**

	**Body perception**					
**Variables**	**Crude analysis RRR (CI 95%)**		**Adjusted analysis RRR (CI 95%)#**		**Adjusted analysis RRR (CI 95%)**&	
	**Thinner**	**Fatter**	**Thinner**	**Fatter**	**Thinner**	**Fatter**
MEN						
**Body mass index z-score change**		*<0.001* ^*a*^		*<0.001* ^*a*^		*<0.001* ^*a*^
≥ +0.5	0.84 (0.63;1.10)	3.63 (2.81;4.68)	0.85 (0.64;1.13)	3.52 (2.71;4.56)	0.86 (0.65;1.14)	3.56 (2.75;4.60)
−0.49 to +0.49	--	--	--	--	--	--
≤ −0.5	1.65 (1.28;2.11)	1.06 (0.77;1.44)	1.65 (1.28;2.13)	1.05 (0.77;1.45)	1.60 (1.24;2.06)	1.05 (0.77;1.44)
WOMEN						
**Body mass index z-score change**		*<0.001* ^*a*^		*<0.001* ^*a*^		*<0.001* ^*a*^
≥ +0.5	0.62 (0.38;1.02)	3.85 (2.86;5.18)	0.51 (0.32;0.91)	4.08 (3.00;5.56)	0.57 (0.35;0.95)	3.71 (2.75;5.01)
−0.49 to +0.49	--	--	--	--	--	--
≤ −0.5	1.18 (0.85;1.64)	0.97 (0.75;1.26)	1.26 (0.89;1.78)	0.96 (0.73;1.26)	1.20 (0.85;1.68)	0.98 (0.75;1.28)

CI: confidence interval ^a^Heterogeneity test ^#^adjusted for confounding variables (maternal schooling, factorial assets index in 1984, and family income in 1982) & adjusted for mediators (having children, marital status, work during the month prior to the interview, psychological well-being, smoking. insufficient physical activity, and presence of minor psychiatric disorders).

Pelotas, Rio Grande do Sul state, Brazil, 1982 to 2004–5.

Following adjustments for possible confounding factors, the associations remained statistically significant. Men and women exhibiting increased BMI z-score between 4 and 23 years of age were less likely to perceive themselves as thinner than ideal (although this finding was only statistically significant for women; RRR = 0.51 95% CI: 0.32 to 0.91), whereas the likelihood of perceiving themselves as fatter than ideal was similar for men (RRR = 3.52 95% CI: 2.71 to 4.56) and women (RRR = 4.08 95% CI: 3.00 to 5.56). For individuals exhibiting decreased BMI between 4 and 23 years of age, the risk of feeling thinner than ideal remained statistically significant only for men (RRR = 1.65 95% CI: 1.28 to 2.13), and the possibility of feeling fatter than ideal was not statistically significant for both men and women (Table 3).

Following the analysis adjusted for confounding factors, an analysis adjusted for mediating factors (behavioral variables) was performed, which revealed the same trends as the previous analysis (Table 3).

In the present study, the weight status at 23 years was not included in the adjusted analyses as a covariate, but alternatively the same adjusted analyses stratified by BMI at 23 years was conducted (data not shown). The associations remained in the same direction.

The findings of the analyses of body perception according to changes in body weight between 18 and 23 years of age, only for men, were in the same direction and stronger taking into account the proximity in time to the outcome.

## Discussion

The present study revealed that individuals with positive BMI z-score changes, i.e., those with increased BMI during the period studied, exhibited higher risks of body dissatisfaction due to perceiving themselves as fatter than ideal. The body image dissatisfaction was higher for women. These results were confirmed by the adjusted analyses.

The results demonstrated that the changes in body weight between 4 and 23 years of age were strongly associated with body dissatisfaction in adulthood and that the prevalence of body dissatisfaction was high for nearly all categories of z-score change for both sexes.

It should be noted that the dissatisfaction in men due to perceiving themselves as thinner than ideal in men exhibiting decreased BMI z-scores or as fatter than ideal in men exhibiting increased BMI z-scores was similar, with dissatisfaction reported in 41% and 48%, respectively. This result confirms the initial hypothesis that individuals perceive themselves as thinner than ideal when there is a decrease in BMI and as fatter than ideal when BMI increases.

The same trend was observed for women, although there was a greater dissimilarity between the reported dissatisfaction; 21% of women who decreased BMI z-scores perceived themselves as thinner than ideal, whereas dissatisfaction was reported in 81.3% of women who increased BMI z-scores perceiving themselves as fatter than ideal. This result also confirmed the initial hypothesis that dissatisfaction due to feeling fatter than ideal is higher in women than in men, which is strongly related to the current beauty standards, which glorify a thin body as ideal.

The adjusted analyses, when adjusted for either confounding or mediating variables, revealed similar dissatisfaction among men and women due to the perception of being thinner or fatter than ideal. However, women whose BMI increased over time became more dissatisfied with their body size and consequently with their body image. The same trend was observed for men, but women reported higher dissatisfaction than men like in the majority of published studies [14-16]. A possible explanation for these findings is that in young adults, the pressures relative to body image (dis)satisfaction are pronounced, with a greater degree of preoccupation with current social standards that tends to stabilize at older ages [33].

Generally, women are more concerned about their weight and body shape. The constant search for the perfect body, widely reported by the media, becomes an increasingly distant goal to be achieved resulting in body dissatisfaction.

Holsen et al. [26] reported a linear increase in body image satisfaction during teenage years, followed by a stabilization during adult life for both sexes. Similarly to the results of the present study, the increase in BMI had an additional negative effect on body image satisfaction in adulthood, resulting in women feeling dissatisfied because they perceived themselves as fatter than ideal.

In the study by Eisenberg et al. [27], body satisfaction decreased in all groups following the five years of follow-up, except for older female teenagers. In general, women who lowered their BMI were more satisfied, and both men and women with increased BMI were less satisfied with their bodies.

That study also revealed that men who were originally eutrophic and lost weight reported a pronounced decrease in body satisfaction, whereas those who normalized from being overweight reported an increase in body satisfaction. These results also were evidenced in the present study, probably related with male’s obsession with muscles [34].

The findings of the present study are important because they demonstrate that young adults exhibiting an increase in BMI from childhood to adulthood are at high risk of body image dissatisfaction at 23 years of age. This dissatisfaction can result in the search for alternative behaviors and unhealthy habits, which are frequently extreme, inadequate, and ineffective [35-38].

The use of the figures for the evaluation of body shape satisfaction is a possible limitation of the present study. Although the use of the body shape figures is controversial among researchers, whether due to the subtle difference between figures, to the use of the same shape to evaluate different biotypes, or to the use of different materials and ways to present the scale, the use of this scale, which has been validated in Brazil [39], was considered adequate due to its practicality, rapid application, and good correlation with BMI [18,40]. Two recent reviews highlight the positive aspects of the figure scale [41,42].

Other possible limitations of the present study are related to the losses inherent in longitudinal studies and the final assessment was conducted some ten years ago. However, it is believed that the results of the present study were not biased because the follow-up rate was not associated with demographic or socioeconomic variables or were in direction to the hypothesis of the study, considering that the prevalence of body dissatisfaction and/or overweight may be increasing in recent years.

Another possible limitation is related to the fact that only the two time points were considered, however the stronger effect of changes in body weight between 18 and 23 years of age in body perception in men suggest that more than two points analyzed could explain better the effects of each period (from childhood to adolescence; from adolescence to adulthood; both) on body perception.

## Conclusions

Individuals exhibiting increased BMI z-scores between 4 and 23 years of age reported higher risks of body dissatisfaction at 23 years of age. Considering the current excess weight indices and the consequent negative effects, it is increasingly evident that healthcare should be delivered in a cross-disciplinary manner, seeking the adequate management of factors related to physiological, psychological, emotional and social processes. It is important to note that in the group exhibiting excess weight, the perception of feeling fatter than ideal may be a positive result because it can drive individuals to healthier habits, whereas for the eutrophic group, dissatisfaction due to perceiving themselves as fatter than ideal deserves increased attention because this group is more predisposed to risky behaviors. Public health policies which implement health promotion and prevention programs, for young people dissatisfied with their weight and body image, become crucial in obtaining better quality of life for this population. Cross-disciplinary healthcare should be prioritized at all life stages according to the inherent peculiarities of each stage.

## References

[1] Coutinho JG, Gentil PC, Toral N (2008). A desnutrição and obesidade no Brasil: o enfrentamento com base na agenda única da nutrição [Malnutrition and obesity in Brazil: dealing with the problem through a unified nutritional agenda]. Cad Saúde Pública.

[2] Ramos FP, Santos LAS, Reis ABC (2013). Educação alimentar e nutricional em escolares: uma revisão de literatura [Food and nutrition education in school: a literature review]. Cad Saúde Pública.

[3] Ministério da Saúde (2012). VIGITEL brasil 2012: vigilância de fatores de risco e proteção para doenças crônicas por inquérito telefônico [surveillance of risk and protective factors for chronic diseases through telephone survey].

[4] Bayer O, Krüger H, von Kries R, Toschke AM (2011). Factors associated with tracking of BMI: a meta-regression analysis on BMI tracking. Obesity (Silver Spring).

[5] Herman KM, Craig CL, Gauvin L, Katzmarzyk PT (2009). Tracking of obesity and physical activity from childhood to adulthood: the physical activity Longitudinal study. Int J Obes Pediatr.

[6] Dietz W (1998). Health consequences of obesity in youth: childhood predictors of adult disease. Pediatrics.

[7] Monteiro PO, Victora CG, Barros FC, Monteiro LMA (2003). Birth size, early childhood growth, and adolescent obesity in a Brazilian birth cohort. Int J Obes.

[8] Starc G, Strel J (2011). Tracking excess weight and obesity from childhood to young adulthood: a 12-year prospective cohort study in Slovenia. Public Health Nutr.

[9] World Health Organization (WHO) (2003). Diet, nutrition and the prevention of chronic diseases. Report of a joint WHO/FAO expert consultation. WHO technical report series, No. 916.

[10] Fogelholm M, Kronholm E, Kukkonen-Harjula K, Partonen T, Partinen M, Härmä M (2007). Sleep-related disturbances and physical inactivity are independently associated with obesity in adults. Int J Obes (Lond).

[11] Must A, Strauss R (1999). Risks and consequences of childhood and adolescent obesity. Int J Obes Relat Metab Disord.

[12] Vartanian LR, Smyth JM, Zawadzki MJ, Heron KE, Coleman SR (2014). Early adversity, personal resources, body dissatisfaction, and disordered eating. Int J Eat Disord.

[13] Stephen EM, Rose JS, Kenny L, Rosselli-Navarra F, Weissman RS (2014). Prevalence and correlates of unhealthy weight control behaviors: findings from the national longitudinal study of adolescent health. J Eat Disord.

[14] Neighbors L, Sobal J (2007). Prevalence and magnitude of body weight and shape dissatisfaction among university students. Eat Behav.

[15] Lynch E, Liu K, Wei GS, Spring B, Kiefe C (2009). The relation between body size perception and change in body mass index over 13 years: the coronary artery risk development in young adults (CARDIA) study. Am J Epidemiol.

[16] Gross SM, Gary TL, Browne CD, LaVeist TA (2005). Gender differences in body image and health perceptions among graduating seniors from a historically black college. J Natl Med Assoc.

[17] Zaragoza JC, Saucedo-Molina TJ, Cortés TLF (2011). Asociación de impacto entre factores socioculturales, insatisfacción corporal, e índice de masa corporal en estudiantes universitarios de Hidalgo, México [Odds ratio between sociocultural factors, body dissatisfaction, and body mass index in university students of Hidalgo, Mexico]. Arch Latinoam Nutr.

[18] Tessmer CS, Silva MC, Pinho MN, Gazalle FK, Fassa AG (2006). Insatisfação corporal em freqüentadores de academia [Body dissatisfaction among gym customers]. Rev Bras Ci Mov.

[19] van den Berg P, Thompson JK, Obremski-Brandon K, Coovert M (2002). The Tripartite Influence model of body image and eating disturbance: a covariance structure modeling investigation testing the mediational role of appearance comparison. J Psychosom Res.

[20] Damasceno VO, Vianna VRA, Vianna JM, Lacio M, Lima JRP, Novaes JS (2006). Imagem corporal e corpo ideal [Body image and ideal body]. R Bras Ci Mov.

[21] Mond J, van den Berg P, Boutelle K, Hannan P, Neumark-Sztainer (2011). Obesity, body dissatisfaction, and emotional well-being in early and late adolescence: findings from the project EAT study. J Adolescent Health.

[22] Westerberg-Jacobson J, Ghaderi A, Edlund B (2012). A longitudinal study of motives for wishing to be thinner and weight-control practices in 7- to 18-year-old Swedish girls. Eur Eat Disord Rev.

[23] Gualdi-Russo E, Vanessa, Manzon S, Masotti S, Toselli S, Albertini A (2012). Weight status and perception of body image in children: the effect of maternal immigrant status. Nutr J.

[24] Bucchianeri MM, Arikian AJ, Hannan PJ, Eisenberg ME, Neumark-Sztainer D (2013). Body Dissatisfaction from Adolescence to Young Adulthood: Findings from a 10-Year Longitudinal Study. Body Image.

[25] Pallan MJ, Hiam LC, Duda JL, Adab P (2011). Body image, body dissatisfaction and weight status in south asian children: a cross-sectional study. BMC Public Health.

[26] Holsen I, Jones DC, Birkeland MS (2012). Body image satisfaction among Norwegian adolescents and young adults: a longitudinal study of the influence of interpersonal relationships and BMI. Body Image.

[27] Eisenberg ME, Neumark-Sztainer D, Paxton SJ (2006). Five-year change in body satisfaction among adolescents. J Psychosom Res.

[28] Victora CG, Barros FC (2006). Cohort profile: the 1982 Pelotas (Brazil) birth cohort study. Int J Epidemiol.

[29] Barros FC, Victora CG, Horta BL, Gigante DP (2008). Metodologia do estudo da coorte de nascimento de 1982 a 2004–5, Pelotas, RS [Methodology of the Pelotas birth cohort study from 1982 to 2004–5, Southern Brazil]. Rev Saude Publica.

[30] World Health Organization (WHO) (2006). Child growth standards: methods and development: length/height-for-age, weight-for-age, weight-for-length, weight-for-height and body mass index-for-Age.

[31] Stunkard AJ, Sörensen T, Schulsiger F, Kety S, Roland L, Sidman R, Matthysse S (1983). Use of the Danish adoption register for the study of obesity and thinness. The genetics of neurological and psychiatric disorders.

[32] Barros AJ, Victora CG (2005). [A nationwide wealth score based on the 2000 Brazilian demographic census]. Rev Saude Publica.

[33] Kuh D, Hardy R (2002). A life course approach to women’s health.

[34] Behar R, Molinari D (2010). [Muscle dysmorphia, body image and eating behaviors in two male populations]. Rev Med Chil.

[35] Latimer LA, Velazquez CE, Pasch KE (2013). Characteristics and behaviors of non-overweight college students who are trying to lose weight. J Prim Prev.

[36] Quick V, Wall M, Larson N, Haines J, Neumark-Sztainer D (2013). Personal, behavioral and socio-environmental predictors of overweight incidence in young adults: 10-yr longitudinal findings. Int J Behav Nutr Phys Act.

[37] Joh HK, Oh J, Lee HJ, Kawachi I (2013). Gender and socioeconomic status in relation to weight perception and weight control behavior in Korean adults. Obes Facts.

[38] Jeffers A, Benotsch EG, Koester S (2013). Misuse of prescription stimulants for weight loss, psychosocial variables, and eating disordered behaviors. Appetite.

[39] Scagliusi FB, Alvarenga M, Polacow VO, Cordás TA, de Oliveira Queiroz GK, Coelho D (2006). Concurrent and discriminant validity of the Stunkard’s figure rating scale adapted into Portuguese. Appetite.

[40] Quadros TMB, Gordia AP, Martins CR, Silva DAS, Ferrari EP, Petroski EL (2010). Imagem corporal em universitários: associação com estado nutricional e sexo [Body image among university students: association with nutritional status and gender]. Motriz (Online).

[41] Moraes C, Anjos LA, Marinho SMSA (2012). Construção, adaptação and validação de escalas de silhuetas para autoavaliação do estado nutricional: uma revisão sistemática da literatura [Development, adaptation and validation of silhouette scales for self-assessment of nutritional status: a systematic review]. Cad Saúde Pública.

[42] Côrtes MG, Meireles AL, Friche AAL, Caiaffa WT, Xavier CC (2013). O uso de escalas de silhuetas na avaliação da satisfação corporal de adolescentes: revisão sistemática da literatura [Silhouette scales and body satisfaction in adolescents: a systematic literature review]. Cad Saúde Pública.

